# Assessing the accuracy and consistency of answers by ChatGPT to questions regarding carbon monoxide poisoning

**DOI:** 10.1371/journal.pone.0311937

**Published:** 2024-11-20

**Authors:** Jun Qiu, Youlian Zhou

**Affiliations:** Chengdu First People’s Hospital Intensive Care Unit, Chengdu, Sichuan, China; Chulalongkorn University, THAILAND

## Abstract

**Background:**

ChatGPT, developed by OpenAI, is an artificial intelligence software designed to generate text-based responses. The objective of this study is to evaluate the accuracy and consistency of ChatGPT’s responses to single-choice questions pertaining to carbon monoxide poisoning. This evaluation will contribute to our understanding of the reliability of ChatGPT-generated information in the medical field.

**Methods:**

The questions utilized in this study were selected from the "Medical Exam Assistant (Yi Kao Bang)" application and encompassed a range of topics related to carbon monoxide poisoning. A total of 44 single-choice questions were included in the study following a screening process. Each question was entered into ChatGPT ten times in Chinese, followed by a translation into English, where it was also entered ten times. The responses generated by ChatGPT were subjected to statistical analysis with the objective of assessing their accuracy and consistency in both languages. In this assessment process, the "Medical Exam Assistant (Yi Kao Bang)" reference responses were employed as benchmarks. The data analysis was conducted using the Python.

**Results:**

In approximately 50% of the cases, the responses generated by ChatGPT exhibited a high degree of consistency, whereas in approximately one-third of the cases, the responses exhibited unacceptable blurring of the answers. Meanwhile, the accuracy of these responses was less favorable, with an accuracy rate of 61.1% in Chinese and 57% in English. This indicates that ChatGPT could be enhanced with respect to both consistency and accuracy in responding to queries pertaining to carbon monoxide poisoning.

**Conclusions:**

It is currently evident that the consistency and accuracy of responses generated by ChatGPT regarding carbon monoxide poisoning is inadequate. Although it offers significant insights, it should not supersede the role of healthcare professionals in making clinical decisions.

## Background

Carbon monoxide (CO) is a colorless, odorless gas produced by the incomplete combustion of carbon-based fuels. Carbon monoxide poisoning remains the leading cause of accidental poisoning deaths worldwide. In the United States, it is estimated that CO poisoning results in approximately 50,000 to 100,000 emergency room visits and 1,500 to 2,000 deaths each year [1, 2]. Moreover, the annual hospital costs associated with CO poisoning exceed $1 billion, as it causes hypoxic damage to the brain and contributes significantly to high mortality rates [3,4]. In China, particularly in rural regions during the winter season, the predominant method of heating is the combustion of coal, which results in a considerable number of hospitalizations each year due to carbon monoxide poisoning. A notable consequence of CO poisoning is CO encephalopathy, which has been evidenced to impair cognitive function, diminish labor productivity, and impose a considerable economic burden on families and society [5]. Furthermore, individuals diagnosed with carbon monoxide toxic encephalopathy require comprehensive rehabilitation and ongoing care. In conclusion, carbon monoxide (CO) poisoning has the potential to result in long-term disability or even death. Fortunately, the occurrence of carbon monoxide poisoning can be prevented through the implementation of efficacious prevention strategies.

As the Internet has expanded, an increasing number of individuals have sought medical-related scientific information from online sources. The advent of new social media platforms has resulted in the emergence of a significant new avenue for the dissemination of health education information [6]. The advancement of artificial intelligence (AI), particularly in the domain of large language models (LLMs), has exhibited a notable acceleration in recent times. OpenAI’s Chatbot Generative Pre-Trained Transformer (ChatGPT) is a notable example, having received considerable global attention upon its release. ChatGPT functions as an intelligent conversational agent, capable of responding to a multitude of inquiries and generating text output that closely resembles that of a human being. It has been utilized extensively across a multitude of medical domains, encompassing a plethora of applications, including clinical decision support, medical inquiries, and medical documentation. The integration of AI tools, such as ChatGPT, into the healthcare sector has the potential to significantly enhance the capacity of both patients and healthcare professionals to access information and support. Firstly, the dissemination of knowledge online provides individuals with the opportunity to enhance their health literacy by acquiring information about health conditions, preventative measures, and treatment options. Furthermore, the integration of AI into the healthcare sector has the potential to enhance patient engagement and empower individuals to assume a more proactive role in their own care. Moreover, the promotion of health literacy via the Internet can facilitate the dissemination of information, thereby reducing disparities in access, particularly in communities that are underserved. In conclusion, the deployment of tools such as ChatGPT to disseminate health literacy has the potential to enhance public health education efforts and, ultimately, improve the health status and quality of life for a significant proportion of the population.

In recent years, a considerable body of research has been conducted to assess the potential applications of ChatGPT in the medical field. The majority of these studies have yielded positive findings, indicating that ChatGPT has the potential to be a valuable tool in this domain. It has been reported that ChatGPT has demonstrated the ability to successfully complete the United States Medical Licensing Examination (USMLE) [7]. Additionally, prior research has demonstrated that ChatGPT attains a consistency rate of 85.4% and an accuracy rate of 57.33% for endodontic inquiries [8]. Moreover, it is noteworthy that ChatGPT has exhibited remarkable accuracy in addressing queries related to benign prostatic hyperplasia and prostate cancer. Its accuracy rates for these topics reached 90% and 94.2%, respectively [9].

To date, no studies have been conducted to evaluate the performance of ChatGPT in answering questions related to carbon monoxide poisoning. The objective of this study was to assess the accuracy and consistency of responses generated by ChatGPT to questions pertaining to carbon monoxide (CO) poisoning.

## Materials and methods

The data for this study were obtained from the "Medical Exam Assistant" (Yi Kao Bang), a widely utilized resource by medical students preparing for the Chinese Medical Licensing Examination and Intermediate Professional Titles. According to statistical data, the Medical Exam Assistant application has been downloaded a total of 3.52 million times since its initial release. The application comprises 44 single-choice questions pertaining to carbon monoxide poisoning. Consequently, a total of 44 questions pertaining to carbon monoxide poisoning were selected. Each item is a single-choice question, with four to five potential responses. The decision to utilize single-choice questions was predicated on a number of considerations, as detailed below. Primarily, the objective was to optimize the scoring methodology and operational procedures. Secondly, in the case of single-choice questions, the correct answer is unique, thereby facilitating a more objective evaluation of the accuracy of the responses generated by ChatGPT.

ChatGPT is a large language model (LLM) powered by artificial intelligence, currently available in two versions: GPT-3.5 and GPT-4. In conducting our analysis, we elected to utilize GPT-3.5 due to its status as a freely available, publicly accessible resource. An observational cohort study was conducted on December 29, 2023. The original Chinese text was entered into ChatGPT ten times for each question. Subsequently, the identical question was translated into English and entered ten times. Following the presentation of the question stem, a prompt indicating the optimal response, namely "Choose the only correct answer based on the stem," will be provided. In consideration of the variability observed in ChatGPT’s responses, the temperature parameter was set to a default value of 0.7. In essence, the temperature parameter controls the randomness of the model’s output, which has a marked effect on the probability distribution used to generate text. Lower temperature settings (e.g., 0.1 to 0.5) result in the generation of responses that are more deterministic and focused, whereas higher settings (e.g., 0.7 to 1.0 and above) lead to greater variability and creativity in the output. The responses from ChatGPT were meticulously documented in an Excel spreadsheet (Microsoft).

### Statistical analysis

#### Accuracy

Each inquiry was entered into GPT with a fixed prompt, and the responses generated by ChatGPT were obtained for each question. Subsequently, the responses were evaluated in comparison to the reference answers provided by the application in order to ascertain their accuracy. In the event of a match between the response generated by ChatGPT and the reference answer, it was regarded as correct; in contrast, if there was a discrepancy, it was deemed incorrect. Then, the percentage of correct responses generated by ChatGPT relative to the total number of questions posed was calculated, thus providing a quantitative measure of its overall accuracy.

#### Consistency

In this study, we employed Shannon entropy [10], a well-established metric for quantifying order or disorder within sequences, as a key measure. In essence, the Shannon entropy is a statistical measure utilized to quantify the degree of information or uncertainty present within a probability distribution. The value of Shannon entropy ranges from 0, which indicates no uncertainty, to 1, which represents maximum uncertainty. The application of Shannon entropy is multifaceted, encompassing a range of disparate fields, including the analysis of DNA sequences and the evaluation of the electroencephalographic effects of desflurane. Moreover, it is utilized in the evaluation of the consistency of ChatGPT in responding to clinical inquiries pertaining to hypertension guidelines [11–13]. In the context of healthcare, the evaluation of the consistency of responses generated by ChatGPT is of particular importance, as accurate and consistent information is essential for patient safety, education, and informed decision-making. Inconsistency has the potential to create confusion or disseminate misinformation, which may ultimately result in adverse health effects. Given the aforementioned factors, this study employs entropy as a metric for evaluating the consistency of ChatGPT responses. In order to calculate the entropy, a set of responses generated by ChatGPT for each question was first constructed by repeating the same question 10 times. Subsequently, the frequency of each unique response was determined, and these frequencies were employed to calculate the entropy for each set of responses. All results were documented in Excel and subsequently subjected to statistical evaluation and graphing using Python.

## Results

**Fig 1:**
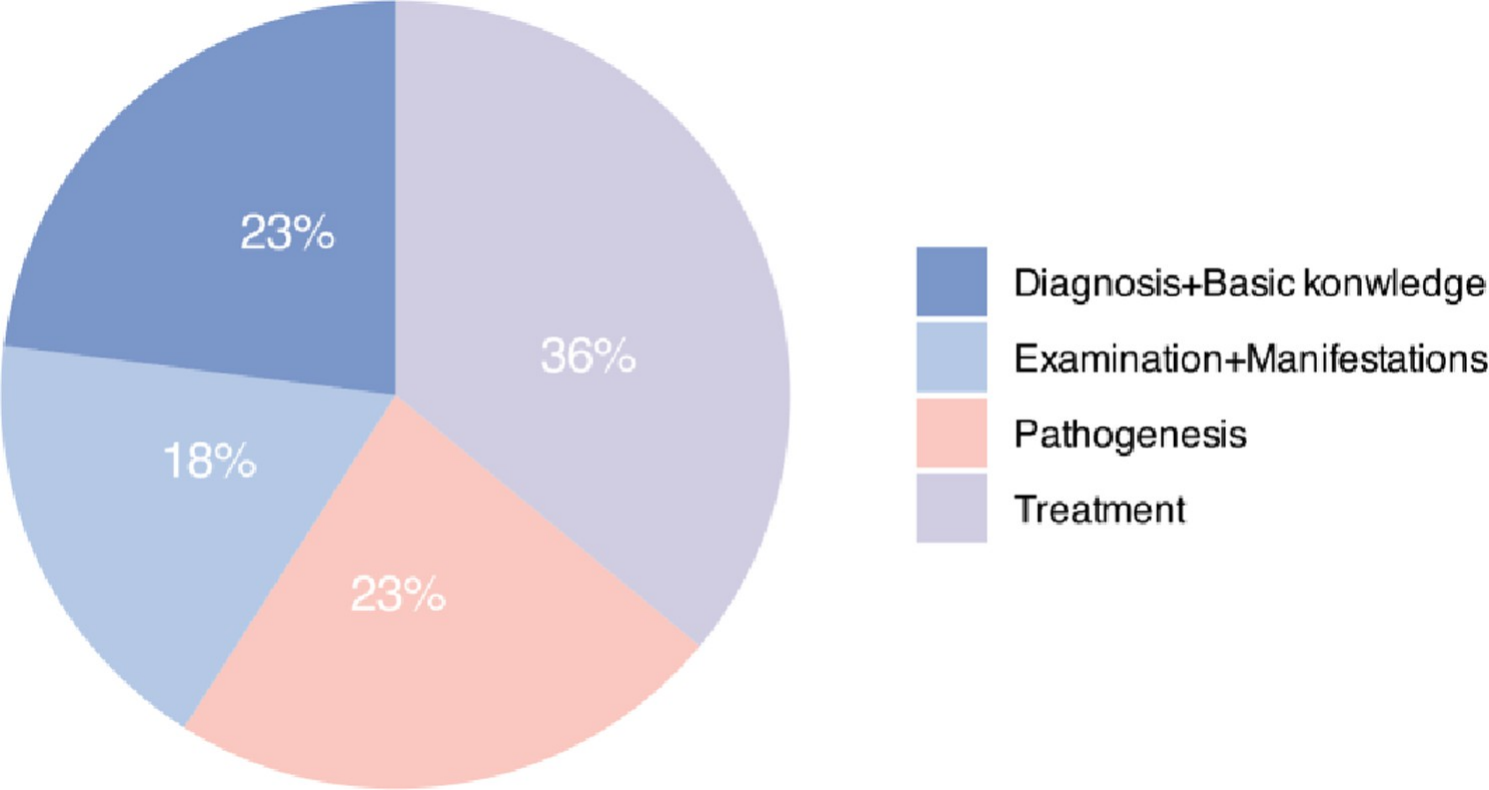
Classification questions.

The analysis encompassed a total of 44 single-choice questions, which were classified into four primary categories based on their content. The categories included Pathogenesis (n = 10, 23%), Examination and Clinical Manifestation (n = 8, 18%), Diagnosis and Basic Knowledge (n = 10, 23%), and Treatment (n = 16, 36%) (see Fig 1).

**Fig 2:**
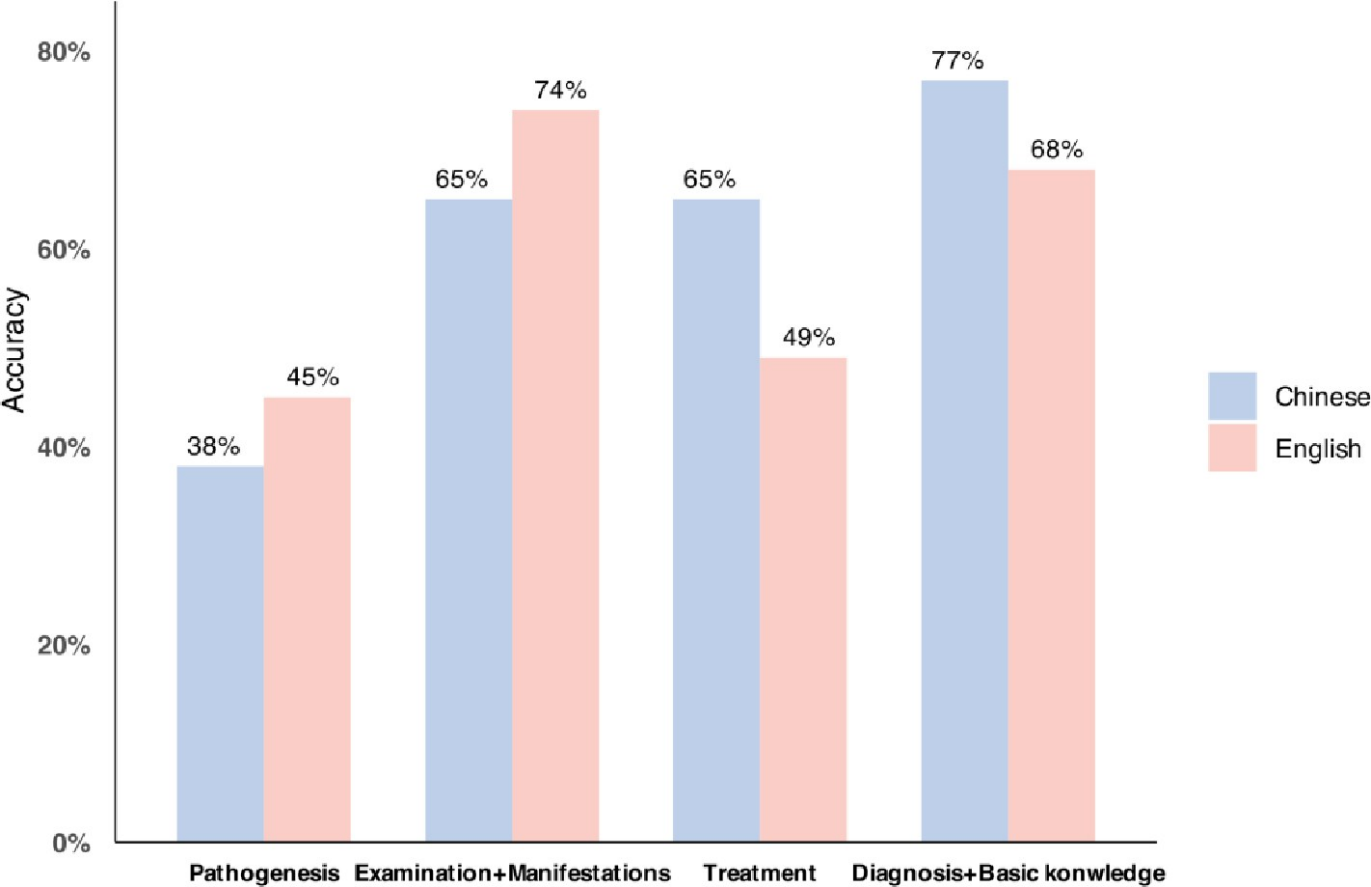
Accuracy in Chinese versus English.

ChatGPT was successful in answering all single-choice questions and provided responses that it considered to be the most appropriate. The overall accuracy rates of the Chinese and English queries exhibited minimal discrepancy, with 61% and 57%, respectively. An analysis of the four primary categories revealed that English responses exhibited a higher degree of accuracy in the Examination and Clinical Manifestations category, with a score of 74% compared to 65% for Chinese responses. In contrast, Chinese responses demonstrated superior accuracy in the domain of diagnosis and basic knowledge, with a rate of 77% compared to 68% for English. In the domain of treatment, Chinese responses exhibited a higher degree of accuracy (65%) compared to English responses (49%). However, both exhibited a low level of accuracy in the pathogenesis category (for further details, please refer to Fig 2).

In order to evaluate the consistency of ChatGPT’s responses to the same questions, we employed the use of Shannon entropy (Table 1). In the English response, 24 of the 44 questions exhibited zero entropy (i.e., the responses were identical), while 15 demonstrated entropy values greater than 0.5 (i.e., the responses exhibited an unacceptable degree of ambiguity). In the Chinese response, 20 responses exhibited zero entropy, while 15 demonstrated entropy values exceeding 0.5.

**Table 1:** Accuracy of answers and Shannon entropy when asking ChatGPT in English and Chinese.

Questions	Chinese Accuracy	Entropy	English Accuracy	Entropy
Q1	0.8	0.72	0.2	0.72
Q2	0.4	0.97	1	0
Q3	1	0	1	0
Q4	0.2	0.72	0.3	0.88
Q5	0.6	0.97	0.6	0.97
Q6	1	0	1	0
Q7	0	0	0	0
Q8	1	0	0.9	0.47
Q9	0.1	0.47	0	0
Q10	0.9	0.47	1	0
Q11	0.7	0.88	0	0
Q12	0.4	0.97	0.9	0.47
Q 13	0.7	0.88	0.6	0.97
Q14	0.1	0.47	0	0
Q15	0	0	0.1	0.47
Q16	0.1	0.47	0.8	0.72
Q17	0.9	0.47	1	0
Q18	0.9	0.47	0.9	0.47
Q19	0.9	0.47	1	0
Q20	0.3	0.88	0.1	0.47
Q21	0.7	0.88	1	0
Q22	0.4	0.97	1	0
Q23	1	0	0	0
Q24	0	0	0.2	0.72
Q25	1	0	0.6	0.97
Q26	1	0	1	0
Q27	1	0	1	0
Q28	1	0	1	0
Q29	0.3	0.88	0.2	0.72
Q30	1	0	1	0
Q31	0	0	0.7	0.88
Q32	1	0	0	0
Q33	1	0	1	0
Q34	1	0	0.3	0.88
Q35	1	0	1	0
Q36	0	0	0	0
Q37	0.9	0.47	0.6	0.97
Q38	0.7	0.88	0.5	1
Q39	0.4	0.97	0.3	0.88
Q40	0.3	0.88	0.6	0.97
Q41	1	0	1	0
Q42	0.9	0.47	0.7	0.88
Q43	0.5	1	0	0
Q44	0	0	0	0

## Discussion

The introduction of ChatGPT has prompted considerable interest globally, with the medical community displaying a notable enthusiasm for the technology [14]. The capacity of this chatbot to furnish expeditious, human-like text responses devoid of advertisements contributes to an enhancement in user engagement. Nevertheless, it is imperative to conduct a comprehensive assessment of ChatGPT’s capabilities before evaluating its potential for clinical applications.

In light of the inherent variability of large language models (LLMs), it was anticipated that discrepancies would be observed in the responses [15]. In the present study, a meticulous and comprehensive analysis was conducted to evaluate the consistency and accuracy of ChatGPT’s responses to clinical questions pertaining to carbon monoxide (CO) poisoning. With respect to consistency, approximately 50% of the items exhibited perfect consistency; however, approximately one-third of the items displayed ambiguous or conflicting responses. In terms of accuracy, the performance of ChatGPT is inadequate. With regard to responses in the Chinese language, the accuracy rate was found to be 61.1%, while in the case of responses in English, the accuracy rate was 57%. The findings indicate that ChatGPT currently lacks the requisite reliability to be regarded as a reliable clinical tool. It is imperative that the accuracy of ChatGPT with respect to medical knowledge be enhanced prior to its deployment in healthcare settings.

The results of the study conducted by Umer and Habib indicate that the acceptable accuracy threshold for medical tools should exceed 90% [16]. Nevertheless, our findings indicate that the accuracy of ChatGPT is approximately 60%, a figure that is consistent with the results of other studies in this field. For example, a study demonstrated that in endodontic inquiries, ChatGPT exhibited an accuracy rate of only 57.33% [16]. Other studies have documented accuracy rates of 79.1% for cirrhosis and 74.0% for hepatocellular carcinoma (HCC), while an accuracy rate of 55.8% has been reported for ophthalmology [14, 17]. Moreover, some studies have defined accuracy in a more comprehensive manner, emphasizing that it is also common for the responses generated by ChatGPT to be partially accurate or to comprise both accurate and incorrect information [18]. The findings suggest that, while ChatGPT displays potential in certain domains, its overall accuracy remains inconsistent and frequently fails to meet the rigorous reliability standards necessary for clinical utilization.

In terms of consistency, approximately one-third of the responses exhibited unacceptable blurring of answers. In a 2023 article entitled "Evaluation of the Accuracy of ChatGPT in Answering Clinical Questions on the Japanese Society of Hypertension Guidelines," the author utilized Shannon entropy as a metric for consistency evaluation. The final results were found to be comparable to those obtained in our study [13]. In the same year, Ana Suárez et al. conducted a study to assess the consistency and accuracy of the ChatGPT in responding to endodontic questions. The results demonstrated that the responses generated by the ChatGPT exhibited a high degree of consistency, with an average score of 85.44% [8]. However, the methodology of this article is disparate from that employed in our own study, therefore direct comparison between the two is not possible. Nevertheless, the application of ChatGPT in the field of healthcare must be conducted in accordance with the highest standards. Accordingly, the methodology for consistency assessment should also be multi-pronged. The evaluation metrics employed in this study, including entropy, can be utilized as benchmarks for the advancement of future AI models. The application of these metrics will assist developers in comprehending the diversity and consistency of model outputs, thereby facilitating the development of more efficient algorithms that diminish uncertainty and enhance the reliability of AI systems.

The user-friendly interface and rapid response times of ChatGPT render it more accessible to the general public in today’s information-driven society. However, a significant concern is the phenomenon of "hallucination," whereby the AI generates erroneous or deceptive information [18, 19]. To mitigate this risk, the research team implemented a straightforward strategy, providing a clear prompt that specified the desired outcome. "Please select the only correct option." The objective was to obtain a singular and precise response, thereby reducing the probability of erroneous answers. Furthermore, the high repetition of each question serves to increase the sample size of the article, which is conducive to both repeatability and accuracy assessment.

It appears that there is still a considerable distance to traverse before ChatGPT can be fully integrated into clinical practice. In the field of healthcare, the accuracy of chatbots is of paramount importance, as even minor errors have the potential to result in significant and irreversible consequences for patients and their families. A further crucial issue is that of reproducibility. The responses generated by ChatGPT are subject to variation depending on the phrasing of the question and may also differ over time. It is thus imperative to take into account the intrinsic constraints and potential hazards associated with the deployment of AI in healthcare. It is vital to pursue ongoing research, evaluation, and refinement to establish a robust foundation for the deployment of artificial intelligence (AI) in medical contexts [20, 21]. To guarantee the safety and reliability of AI applications, it is imperative to exercise close supervision, establish fundamental guidelines regulating their utilization, and implement continuous monitoring of AI outputs. This is of paramount importance for the maintenance of the high standards of clinical application.

## Conclusion

The results of our investigation indicate that the accuracy and consistency of ChatGPT’s responses to queries pertaining to carbon monoxide poisoning are suboptimal. This underscores the necessity for meticulous and rigorous assessment when seeking medical counsel from ChatGPT. In conclusion, this study establishes the foundation for the broader implementation of AI technologies in healthcare and delineates the prospective trajectory of technological advancement.

### Limitations

It is essential to recognize that the scope of this study does not encompass comprehensive clinical inquiries. Instead, the study focuses on single-choice questions, which may introduce a certain degree of response bias into the results. Secondly, it should be noted that the AI model is subject to continual updates, which may result in varying responses depending on the timing and context of the question.

### Strengths

On a positive note, AI offers convenient and expedient access to information, particularly in remote locations and regions that lack adequate healthcare infrastructure. It is capable of supporting multiple languages and facilitates natural communication through platforms such as ChatGPT. It is of paramount importance that artificial intelligence models undergo continuous updates and iterations, in alignment with the advancements in technology. The incorporation of AI into real-time clinical databases and hospital systems has the potential to significantly improve healthcare efficiency and reduce the workload of clinicians. The implementation of this integration, in conjunction with the maintenance of accuracy and consistency, will not only enhance physicians’ clinical decision-making abilities but will also lead to improved patient outcomes.

## Supporting information

S1 Fig (R)

S2 Fig (R)

S1 Data (XLSX)

S2 Data (XLSX)
